# DRG payment does not predispose to negative clinical outcomes in general surgery cases: evidence from a tertiary hospital in China

**DOI:** 10.3389/fpubh.2025.1614647

**Published:** 2025-09-29

**Authors:** Rui Hou, Xiaokun Liu, Weijie Chen, Xutong Tan, Zhe Li, Weiguo Zhu, Weibin Wang

**Affiliations:** ^1^Department of General Surgery, Peking Union Medical College Hospital, Beijing, China; ^2^Department of Medical Insurance Management, Peking Union Medical College Hospital, Beijing, China; ^3^Department of Medical Affairs, Peking Union Medical College Hospital, Beijing, China; ^4^Department of Information Management, Peking Union Medical College Hospital, Beijing, China; ^5^Department of Primary Care and Family Medicine, Peking Union Medical College Hospital, Beijing, China

**Keywords:** DRG, general surgery, negative clinical outcome, unplanned reoperation, in-hospital mortality, readmission

## Abstract

**Background:**

Diagnosis-related group (DRG) payment has proven effective in improving efficiency and containing medical costs. However, concerns persist regarding its potential negative impact on healthcare quality. This study aimed to investigate the association of DRG payment with negative clinical outcomes in general surgery cases.

**Methods:**

The study utilized clinical and insurance data from patients undergoing elective general surgeries at authors' institution between March 2019 and February 2025, with the DRG payment officially implemented in March 2022. Changes in average costs and length of stay (LOS) before and after the DRG payment were assessed using *t*-tests. To evaluate the reform's impact on healthcare quality, interrupted time series analysis (ITSA) was applied to examine changes in the rates of five negative clinical outcomes: in-hospital mortality, unplanned readmission within 31 days, red blood cell transfusion exceeding 10 units, LOS exceeding 30 days, and unplanned reoperations.

**Results:**

The sample included 38,014 discharged cases, including 18,666 cases before and 19,348 cases after DRG implementation. Five groups with the highest case volumes were KD1 (thyroid surgery), GB2 (major operation of intestines and colorectum), HC2 (cholecystectomy), GB1 (major operation of stomach and duodenum), HB1 (major operation of pancreas and liver). Following DRG payment, significant reduction in costs was observed in GB2 and GB1, while significant reduction in LOS was observed in groups GB2, HC2 and HB1. ITSA revealed no significant changes in level or trend for any of the five negative clinical outcomes, either in the overall sample or in the subgroups.

**Conclusion:**

For patients undergoing general surgeries, DRG payment promoted efficiency without increasing the risk of negative clinical outcomes.

## 1 Introduction

DRG (Diagnosis-Related Group) is a case classification system that groups patients with similar clinical characteristics and resource consumption based on factors such as diagnosis, severity, procedures, comorbidities, and other clinical parameters (1). Patients within the same DRG are considered to be medically and economically comparable, forming the basis of a new payment methodology in which hospitals are reimbursed with a standardized fee per group, reflecting the national average cost for patients in that category (2). DRG payment has been proven effective in neutralizing the negative effects of the traditional fee-for-service model, increasing transparency and hospital productivity, and encouraging medical institutions to deliver consistent services at lower costs (3, 4). As a result, it has been widely adopted in the U.S., Europe and some developing countries in Asia to contain the surging medical expenses (1, 5).

Medical services under DRG payment can be evaluated from two key perspectives: efficiency and quality (6). By providing a fixed payment for each patient, the system incentivizes healthcare providers to limit the services offered per patient and to treat more in-patients. These measures effectively reduce hospitalization costs and length of stay (LOS), thereby promoting efficiency (6, 7). However, the quality and equality of the healthcare service may be subject to impairment because, under financial pressure, hospitals may risk undertreating patients (service cuts), discharging them prematurely (bloody discharge), upcoding (DRG creep) or selectively admitting patients based on their potential profitability (cherry picking) (2, 8, 9). Some studies have shown that DRG payment is associated with increased readmission and in-hospital mortality at national or municipal levels, particularly among older adults patients and those with complex comorbidities, while others found no compelling evidence of increased negative clinical outcomes (10, 11). Moreover, some scholars have argued that the implementation of DRG payment contributes to the quality improvement of medical services (12, 13). Given the inconsistency in existing evidence from Western countries, the impact of DRG payment on healthcare quality remains unclear and controversial.

**Figure 1:**
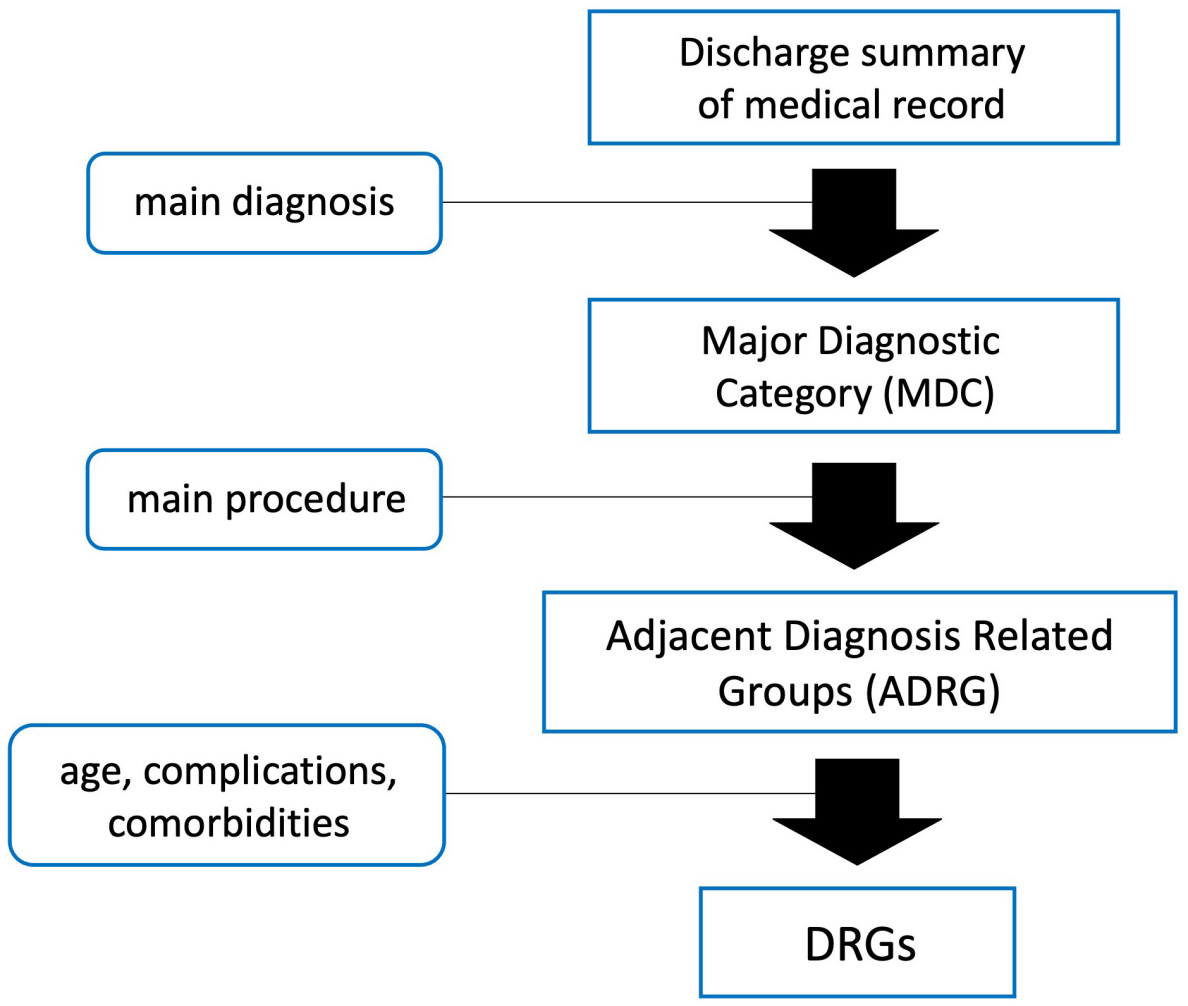
Basic grouping principle of China's DRG system.

Combining Western models with domestic context and data, China officially launched its nationwide DRG system in March 2022. Inpatients are first categorized into Adjacent Diagnosis Related Groups (ADRG) according to their primary diagnosis and main treatment approach. They are then further grouped into specific DRGs based on age, complications and comorbidities. This ensures that cases within the same DRG share similar clinical characteristics and resource consumption (Figure 1) (14). The payment standard for each DRG is determined by multiplying the unit cost by the group's relative weight (RW), which reflects the relative level of hospitalization cost among different DRG groups (14). To understand the clinical implications of DRG payment 3 years after its implementation, we conducted interrupted time series analyses (ITSA) to evaluate changes in negative clinical outcomes before and after the policy was introduced. Five key indicators of negative clinical outcomes were collected and analyzed: in-hospital mortality, unplanned readmission within 31 days, red blood cell (RBC) transfusions exceeding 10 units, LOS exceeding 30 days, and unplanned reoperations. This study aims to provide insights into the impact of DRG payment on medical practice and healthcare quality from the perspective of clinical specialists, and to offer valuable references for policymakers overseeing healthcare payment reforms, particularly in low- and middle-income countries (LMICs).

## 2 Methods

This study followed the Strengthening the Reporting of Observational Studies in Epidemiology (STROBE) reporting guideline (15).

### 2.1 Data sources and study variables

This retrospective cohort study used medical records and insurance settlement information of inpatients who were admitted and received elective surgery from Mar. 2019 to Feb. 2025 at the Department of General Surgery of author's institution. Patients with high-risk mortality, like emergency cases and those with end-stage tumors were excluded because their clinical outcomes are primarily determined by the underlying disease rather than the healthcare quality. The data types included admission date, age, gender, diagnoses, procedure received, LOS, DRG grouping, hospitalization cost. The data were jointly provided by the Department of Medical Affairs, the Department of Medical Insurance and the Department of Medical Records. Preprocessing was conducted to address missing or invalid data fields and to ensure accuracy of medical records and DRG grouping.

Five outcome variables were studied to determine the effect of DRG payment on healthcare quality: in-hospital mortality, unplanned reoperations, unplanned readmission within 31 days after discharge, red blood cell (RBC) transfusions exceeding 10 units within one admission, and LOS exceeding 30 days. To ensure the validity of the in-hospital mortality rate, patients with life-threatening surgical complications—where no treatment can reverse the dying process—are still recorded as in-hospital deaths, even if they are discharged to palliative care based on decisions by physicians or family members. Multiple unplanned reoperations on the same patient during hospitalization were counted separately. Readmissions for reasons unrelated to the initial admission, as well as pre-surgical RBC transfusions exceeding 10 units, were not considered negative outcomes, as they are predominantly associated with patients' health status rather than the quality of healthcare. Other variables including patient volume, CMI (case mix index), average hospitalization costs (presented in Chinese Yuan, CNY), and LOS were also analyzed before and after DRG payment implementation.

### 2.2 Outcomes

Patient-level data on LOS and cost, along with bi-monthly rates of five negative clinical outcomes: in-hospital mortality, unplanned readmission within 31 days, RBC transfusions exceeding 10 units, LOS exceeding 30 days, and unplanned reoperations.

### 2.3 Statistical analysis

The average hospitalization cost and LOS before and after the implementation of DRG payment were compared using a *t*-test. The impact of DRG payment on the rate of each negative clinical outcome, calculated bi-monthly, was assessed using the ITSA method. The ITSA model was specified as follows:

*Y*_*t*_ = *β0* + *β1* × *T* + *β2* × *X*_*t*_ + *β3* × *T* × *X*_*t*_ + *ε*_*t*_,

where *Y*_*t*_ represents the rate of the negative outcome at time point *t*. β_0_ denotes the baseline level at *t* = 0, β*1* is the monthly slope (trend) of the rate before DRG payment, β*2* and β*3* indicate the step change and slope change in the rate after the payment reform, respectively. *T* is the bi-monthly time variable during the study period, and *X*_*t*_ is a dummy variable that equals 0 before the payment reform and 1 after the reform. Based on the study period and the timing of DRG payment initiation, March 2022 was set as the intervention point, with 18 entries both before and after the intervention. ε_*t*_ represents the random errors. Regression model fitting was performed using ordinary least squares segmentation, including tests for normality, homoscedasticity, and adjustments for serial autocorrelation via the Prais-Winsten estimation and the Durbin-Watson statistic (16). ITSA was also conducted for specific ADRGs if the studied negative clinical outcome occurred more than 50 times. The threshold for statistical significance was set at 5%. All analyses were conducted using Stata 16.0 and GraphPad 10.

## 3 Results

### 3.1 Sample characteristics

A total of 38,014 discharged cases were identified to meet our inclusion criteria, including 18,666 cases before DRG payment and 19,348 cases after its implementation. Detailed information including cost, LOS, number and rate of negative clinical outcomes were listed in Table 1. Five ADRGs with the highest case volume were KD1 (thyroid surgery), GB2 (major operation of intestines and colorectum), HC2 (cholecystectomy), GB1 (major operation of stomach and duodenum), HB1 (major operation of pancreas and liver), with their volume and ranking changed after the payment reform. The definition, RW and payment of each ADRG were showed in Table 2.

**Table 1:** Sample characteristics 2019.3–2025.2.

**Variable**	**Before 2019.3–2022.2**	**After 2022.3–2025.2**
Number of patients	18,666	19,348
Number of DRGs	199	199
CMI^a^	1.58	1.68
Top 5 ADRGs with mostpatients (number of cases)	KD1 (9,343)	KD1 (8,600)
	GB2 (3,092)	GB2 (3,620)
	HC2 (1,740)	HC2 (1,257)
	GB1 (914)	HB1 (1,234)
	HB1 (896)	GB1 (1,215)
Average cost (CNY), mean (SD)	27,239 (24,980)	33,667 (28,957)
LOS (d), mean (SD)	7.6 (6.2)	8.2 (7.0)
**Negative clinical outcome (n, %)**		
In-hospital mortality	16, 0.0857%	13, 0.0672%
Unplanned reoperation	185, 0.991%	192, 0.992%
Unplanned readmissionwithin 31 days	142, 0.761%	130, 0.672%
LOS > 30 d	216, 1.157%	225, 1.163%
RBC transfusion > 10 U	69, 0.3697%	68, 0.3515%

^a^Case mix index, = ∑RW of each case/total number of the cases. A metric used to measure the complexity and resource intensity of a hospital's caseload. A higher CMI indicates that the hospital is treating more complex or resource-intensive cases.

**Table 2:** Definition, RW and payment of top 5 ADRGs in terms of number.

**ADRG**	**Definition**	RW^*^	**Payment (CNY)**
KD1	Thyroid surgery	1.09	22,266
GB2	Major operation of intestines and colorectum	4.26	87,110
HC2	Cholecystectomy	0.9	18,382
GB1	Major operation of stomach and duodenum	5.47	111,724
HB1	Major operation of pancreas and liver	4.73	96,648

^*^RW, The relative weight of a DRG based on its cost, reflecting the resource consumption of that DRG compared to the others. An RW value of 1 for a DRG indicates that the average hospitalization cost for that group is equal to the average for all diseases.

### 3.2 Sample characteristics stratified by ADRG

**Figure 2:**
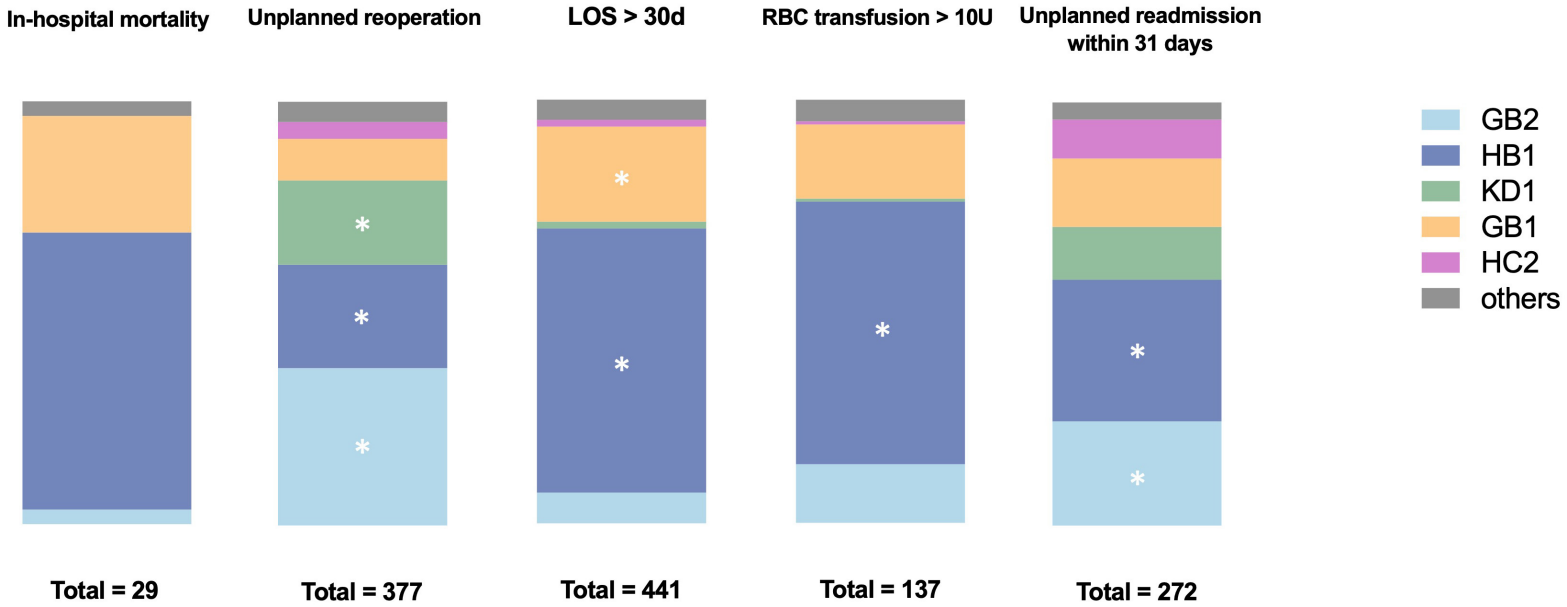
Frequency and composition ratio of each negative clinical outcome. ADRGs with the studied negative clinical outcome occurred more than 50 times were marked with *.

Comparisons of sample characteristics across the top 5 ADRGs were conducted to evaluate the impact of DRG payment reform on specific disease group (Table 3). The analysis revealed a statistically significant increase in hospitalization cost and LOS exclusively within the KD1 cohort. All the other ADRG groups exhibited either notable reductions or stability in these two metrics, which are key indicators of healthcare efficiency. Specifically, a significant reduction in costs was observed in GB2 and GB1, while a significant reduction in LOS was noted in GB2, HC2, and HB1. Figure 2 shows the frequency and composition ratio of each negative clinical outcome.

**Table 3:** Sample characteristics of the top 5 ADRGs 2019.3–2025.2.

**ADRG**	**Variable**	**Before 2019.3–2022.2**	**After 2022.3–2025.2**	***t***
**KD1**	Number of patients	9,343	8,600	-
	Cost (CNY), mean (SD)	15,656 (5,157)	17,049 (6,980)	15.28^***^
	LOS (d), mean (SD)	4.97 (2.0)	5.3 (3.0)	8.73^***^
	**Negative clinical outcome**, ***n*** **(%)**			
	In-hospital mortality	0 (0%)	0 (0%)	-
	Unplanned reoperation	32 (0.34%)	43 (0.50%)	-
	Unplanned readmission within 31 days	20 (0.21%)	14 (0.16%)	-
	LOS > 30 d	4 (0.04%)	3 (0.03%)	-
	RBC transfusion > 10 U	0 (0%)	1 (0.01)	-
**GB2**	Number of patients	3,092	3,620	-
	Cost (CNY), mean (SD)	48,423 (12,375)	46,983 (11,956)	4.84^***^
	LOS (d), mean (SD)	11.03 (5.0)	9.67 (4.28)	12.01^***^
	**Negative clinical outcome**, ***n*** **(%)**			
	In-hospital mortality	0 (0%)	1 (0.03%)	-
	Unplanned reoperation	72 (2.33%)	68 (1.88%)	-
	Unplanned readmission within 31 days	37 (1.20%)	30 (0.83%)	-
	LOS > 30 d	14 (0.45%)	18 (0.50%)	-
	RBC transfusion > 10 U	11 (0.36%)	8 (0.22%)	-
**HC2**	Number of patients	1,740	1,257	-
	Cost (CNY), mean (SD)	12,347 (3,246)	12,546 (3,515)	1.59
	LOS (d), mean (SD)	5.22 (2.64)	4.64 (1.98)	6.56^***^
	**Negative clinical outcome**, ***n*** **(%)**			
	In-hospital mortality	0 (0%)	0 (0%)	-
	Unplanned reoperation	9 (0.52%)	6 (0.48%)	-
	Unplanned readmission within 31 days	17 (0.98%)	8 (0.64%)	-
	LOS > 30 d	4 (0.23)	3 (0.47)	-
	RBC transfusion > 10 U	0 (0%)	1 (0.08%)	-
**HB1**	Number of patients	896	1,234	-
	Cost (CNY), mean (SD)	95,900 (32,077)	97,291 (34,906)	0.91
	LOS (d), mean (SD)	19.87 (9.01)	17.97 (7.98)	5.14^***^
	**Negative clinical outcome**, ***n*** **(%)**			
	In-hospital mortality	10 (1.11%)	9 (0.73%)	-
	Unplanned reoperation	46 (5.13%)	46 (3.73%)	-
	Unplanned readmission within 31 days	37 (4.13%)	54 (4.38%)	-
	LOS > 30 d	134 (14.96%)	141 (11.43%)	-
	RBC transfusion > 10 U	38 (4.24%)	47 (3.81%)	-
**GB1**	Number of patients	914	1,215	-
	Cost (CNY), mean (SD)	71,008 (27,241)	68,530 (26,089)	2.13^*^
	LOS (d), mean (SD)	14.01 (6.07)	13.76 (5.90)	0.96
	**Negative clinical outcome**, ***n*** **(%)**			
	In-hospital mortality	5 (0.55%)	3 (0.25%)	-
	Unplanned reoperation	15 (1.64%)	22 (1.81%)	-
	Unplanned readmission within 31 days	27 (2.95%)	17 (1.40%)	-
	LOS > 30 d	49 (5.36%)	50 (4.12%)	-
	RBC transfusion > 10 U	17 (1.86%)	7 (0.58%)	-

^*^p < 0.05,

^**^p < 0.01,

^***^p < 0.001.

### 3.3 ITSA results of negative clinical outcomes for overall cases and specific ADRGs

**Figure 3:**
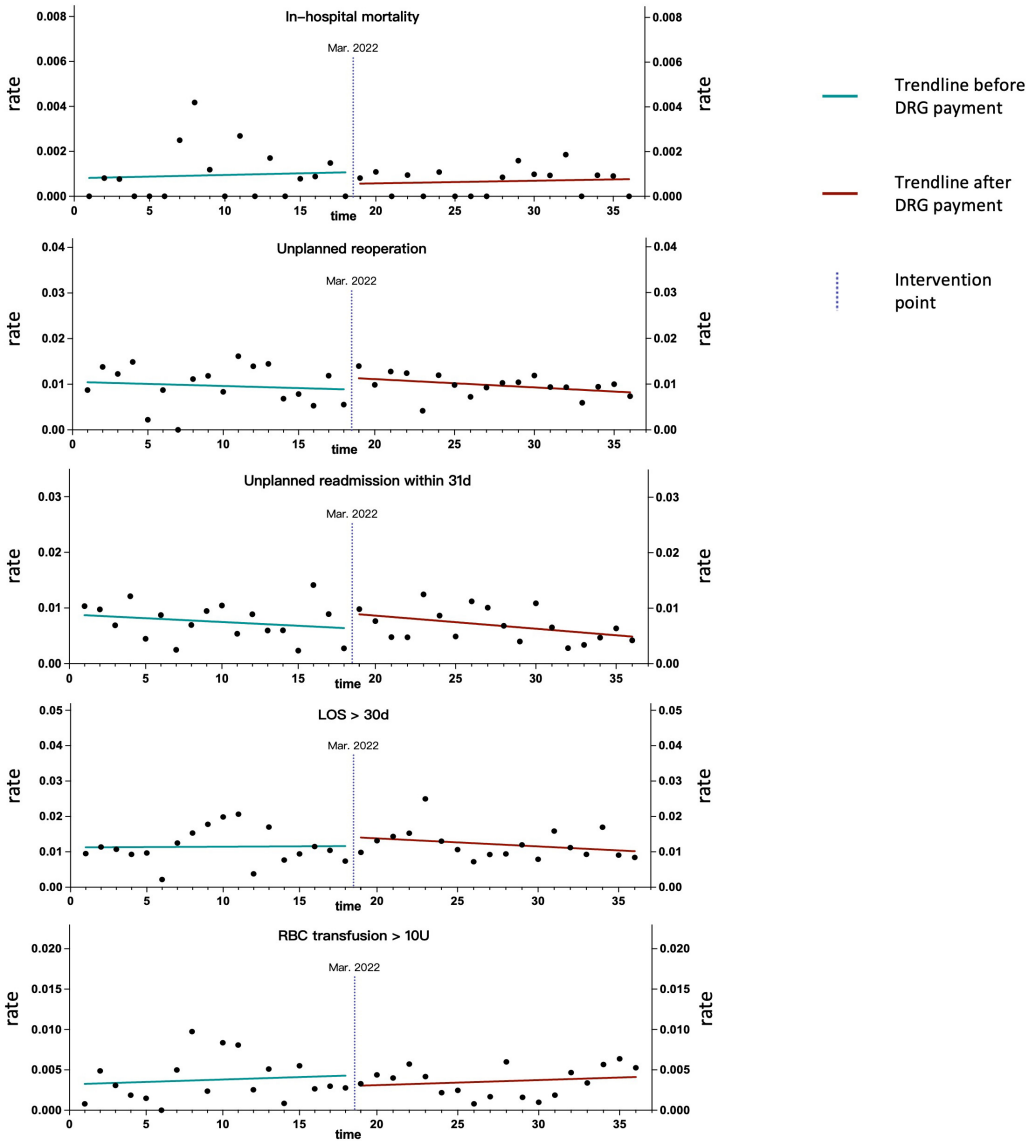
Trend line graphs illustrating the rates of negative clinical outcomes for the overall sample.

**Figure 4:**
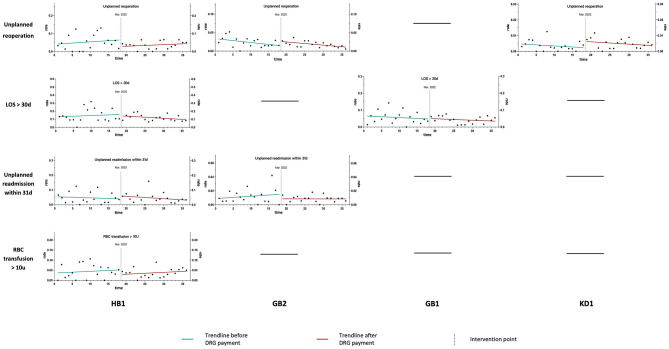
Trend line graphs illustrating the rates of negative clinical outcomes for specific ADRGs.

The baseline slope, step change, and monthly slope change of negative clinical outcomes for the overall sample, as well as for ADRGs with more than 50 occurrences of the studied negative clinical outcomes, are presented in Table 4. Trend line graphs in Figures 3, 4 illustrate the rates of negative clinical outcomes for the overall sample and specific ADRGs, respectively. Prior to the implementation of the DRG payment, neither the overall sample nor the specific ADRGs exhibited a significant trend (non-zero slope) in the rates of negative clinical outcomes. Following the DRG payment, no significant changes were observed in either the level or trend of negative clinical outcomes for the overall sample or any of the examined ADRGs. After the intervention point, the rates of in-hospital mortality and RBC transfusions > 10 units showed a slight upward trend, while the rates of other negative outcomes exhibited a downward trend (Figure 3). All regression models passed the tests for normality and homoscedasticity.

**Table 4:** ITSA results of 5 negative clinical outcomes for overall cases and specific ADRG.

**Negative clinical outcome**		**Baseline monthly slope (**β**1)**		**Step change (**β**2)**		**Monthly slope change (**β**3)**	
		**Estimate (95%CI)**	***p***	**Estimate (95%CI)**	***p***	**Estimate (95%CI)**	***p***
In-hospital mortality	Overall	0.0000 (−0.0001, 0.0001)	0.68	−0.0005 (−0.0013, 0.0003)	0.19	0.0000 (−0.0001, 0.0001)	0.95
Unplanned reoperation	Overall	−0.0001 (−0.0004, 0.0002)	0.56	0.0025 (−0.0015, 0.0065)	0.23	−0.0001 (−0.0004, 0.0002)	0.59
	KD1	−0.0001 (−0.0004, 0.0001)	0.25	0.0040 (−0.0003, 0.0082)	0.07	0.0000 (−0.0004, 0.0004)	0.93
	HB1	0.0013 (−0.0013, 0.0039)	0.33	−0.0368 (−0.0791, 0.0055)	0.09	−0.0004 (−0.0032, 0.0024)	0.78
	GB2	−0.0010 (−0.0023, 0.0002)	0.08	0.0118 (−0.0022, 0.0257)	0.09	0.0002 (−0.0012, 0.0015)	0.81
LOS > 30 d	Overall	0.0000 (−0.0005, 0.0005)	0.92	0.0024 (−0.0047, 0.0095)	0.50	−0.0003 (−0.0010, 0.0004)	0.46
	HB1	0.0015 (−0.0061, 0.0092)	0.68	−0.0188 (−0.1260, 0.0884)	0.72	−0.0043 (−0.0155, 0.0069)	0.44
	GB1	−0.0011 (−0.0034, 0.0012)	0.34	0.0027 (−0.0318, 0.0373)	0.87	0.0002 (−0.0030, 0.0035)	0.88
Unplanned readmission within 31 d	Overall	−0.0001 (−0.0004, 0.0002)	0.33	0.0026 (−0.0017, 0.0070)	0.24	−0.0001 (−0.0004, 0.0002)	0.57
	HB1	−0.0007 (−0.0033, 0.0019)	0.59	0.0189 (−0.0199, 0.0578)	0.33	−0.0009 (−0.0045, 0.0028)	0.62
	GB2	0.0003 (−0.0007, 0.0013)	0.51	−0.0068 (−0.0213, 0.0078)	0.36	−0.0003 (−0.0014, 0.0007)	0.50
RBC transfusion> 10 u	Overall	0.0001 (−0.0001, 0.0002)	0.49	−0.0013 (−0.0038, 0.0012)	0.30	0.0000 (−0.0024, 0.0024)	0.98
	HB1	0.0007 (−0.0015, 0.0029)	0.51	−0.022 (−0.0055, 0.0010)	0.17	0.0002 (−0.0028, 0.0033)	0.87

## 4 Discussion

In traditional fee-for-service model, medical institutions tend to provide more services than actually needed to maximize their income, leading to wasted medical resources and unjustified increases in healthcare costs (2, 17). By introducing a fixed payment per patient, the DRG system incentivizes healthcare providers to limit the services and treat more in-patients for profit. This efficiency-driven mechanism results in reduced hospitalization costs and shortened LOS, as consistently demonstrated by studies from both developed countries and LMICs (1, 10, 13). Our study observed the same effect in groups GB2, HC2, GB1, and HB1, with significant decreases in either LOS or cost. However, in group KD1, an increase in cost and LOS was noted. Further investigation revealed that the post-reform cohort is associated with higher proportion of nodal metastasis and lateral neck dissection, which are more complex and resource-intensive compared to simple lobectomy with or without central node dissection (Supplementary Table S1). This change in patient composition explains the paradoxical increase in LOS and cost for the KD1 group.

On the other hand, DRG payment may lead to negative consequences if its efficiency-promoting effects are so strong that the manipulation of system occurs at three levels: corporate, clinical, and coding (2, 7, 18). These consequences include practices such as cherry picking, upcoding and inferior quality of healthcare (2). Cherry picking refers to selecting patients based on their profitably, upcoding refers to add additional diagnoses or procedures to medical records to place patients in higher paying group—both of which are difficult to detect. Doctors tend to describe medical conditions as more complex and surgeries as more challenging to showcase their skills and achieve a greater sense of professional accomplishment, while the actual condition of the patients is often difficult to verify (19). It is also difficult to determine whether the changes in the patient composition of a medical institution are caused by cherry-picking or shifts in the population's disease spectrum over a certain period. Therefore, our study concentrated on the potential impairment of DRG payment on healthcare quality, as metrics such as in-hospital mortality and readmission rates provide a precise and reliable basis for evaluation.

The impact of DRG payment on healthcare quality has been much less studied than its effect on efficiency. Existing researches, mainly from developed countries, presents highly inconsistent findings, with evidence of improvements, reductions, and no significant changes in service quality coexisting (2, 12, 13, 20). Some studies suggest that a fixed payment for each treated case increases the financial risk for medical institutions, encouraging behaviors such as cutting services, substituting expensive originals with cheaper generics, reducing the use of new technologies, and early discharge, all of which may compromise the quality of healthcare (14, 20, 21). But the existing studies have the following limitations: Data for quality assessment are drawn from the entire population, covering a wide range of diseases and hospitals at different levels, which introduces significant heterogeneity and compromises the accuracy of the findings; Only two metrics, in-hospital mortality and readmission, are used to assess healthcare quality, which does not fully capture the clinical landscape; The studies cover a relatively short timeframe, failing to capture the long-term impacts of the DRG reform. To address these issues, we utilized 6-year data from a single surgical department with a focused range of diseases, and incorporated five negative clinical outcomes as quality assessment metrics. This approach aims to enhance accuracy and minimize heterogeneity in the findings.

As an indicator of healthcare quality, in-hospital mortality is generally rare and shows significant variation across different institutions and departments, which may not fully reflect the overall quality of healthcare or the level of the institution (22). Readmission serves as a metric to evaluate premature discharge or “bloody discharge,” which refers to discharging patients before they meet the discharge criteria in order to treat more patients for profit. In addition to these two traditional indicators, we have incorporated three more indicators—unplanned reoperation, RBC transfusion > 10 U, and LOS > 30 days—all of which are closely associated with the quality of surgery and perioperative management. Before the implementation of the DRG payment, the rates of negative clinical outcomes in the overall sample and specific ADRGs remained relatively stable. This indicates that the institution is proficient in providing standardized and homogeneous healthcare services, delivering consistent treatment outcomes for patients requiring general surgical procedures. More importantly, our study demonstrated that the implementation of DRG payment did not lead to an increased rate in any of these 5 negative clinical outcomes. Individual analyses of each ADRG yielded the same results, further enhancing the reliability of our findings. Notably, following the payment reform, the rates of RBC transfusion > 10 U and in-hospital mortality exhibited a slight upward trend, although not statistically significant. This is because, in the context of the Chinese government's strong promotion of hierarchical medical systems, our department has gradually admitted more complex and high-risk surgical patients (characterized by high RW values, such as GB1, HB1, and GB2) in recent years. Meanwhile, the number of routine surgeries, like thyroid and gallbladder procedures, has significantly decreased. In any case, the quality-neutral effect of DRG payment depends on a well-functioning grouping scheme, accurate payment standards and a robust auditing system. If the payment fails to fully cover the actual resource consumption required for treating the disease and imposes significant financial pressure on institutions, healthcare quality may be compromised (2, 3, 8).

Our study has several limitations. The study period encompasses the initial wave of the COVID-19 epidemic in China. Although cases directly related to COVID-19 were excluded and all negative clinical outcomes were evaluated by rate rather than absolute numbers, some degree of deviation remains. This is because during that time which lasted approximately 4 months, all elective surgeries were delayed and restricted to prioritize the treatment of COVID-19 patients. To address this problem, we have made multi-linear regression for each negative clinical outcomes which demonstrated the non-significant influence of the epidemic. Second, the transmission of health policy to medical institutions and personnel takes time, potentially years. There may be time lags between the implementation of DRG in China and any resulting changes in patient outcomes. The long-term effects of the policy warrant further monitoring and exploration. Additionally, the study used data from a single institution and specifically focused on general surgery cases, which cannot fully reflect the impact of DRG payment on a national scale. However, as one of the leading public hospitals in China, our data demonstrated that high-quality healthcare can be delivered under restricted payment and rapid bed turnover, provided that the treatment trajectory aligns with current clinical guidelines. We believe our experiences may serve as a model for other medical institutions, particularly those in LMICs, to better adapt to this payment reform. To comprehensively understand the full range of potential negative consequences associated with DRG payment, further research is needed to measure the extent of upcoding, cherry-picking, and dumping—behavioral changes that are difficult to assess through conventional quantitative outcomes.

## 5 Conclusion

DRG payment has proven effective in enhancing efficiency, as shown by reduced average costs and LOS. However, concerns remain regarding whether these efficiency gains come at the expense of healthcare quality. Our study demonstrated that, for patients undergoing general surgeries, DRG payment did not increase the rate of negative clinical outcomes while promoting efficiency. Accurate grouping schemes and appropriate payment standards tailored to local context, as well as ongoing audit mechanisms are essential to ensure this quality-neutral effect is achieved on a national scale. As value-based medicine is being phased in, mechanisms for healthcare quality assessment and assurance should be integrated into the reimbursement system (23, 24).

## Data Availability

The data analyzed in this study is subject to the following licenses/restrictions: the data are not publicly available due to privacy. Requests to access these datasets should be directed to the first author (henrequelme@aliyun.com).
