# Supplementary material for: DRG payment does not predispose to negative clinical outcomes in general surgery cases: evidence from a tertiary hospital in China
Source: Front Public Health. 2025 Sep 29;13:1614647. doi: 10.3389/fpubh.2025.1614647 (PMC12516358; doi:10.3389/fpubh.2025.1614647)
Supplement: Supplementary file 1 [file Table_1.docx]

**Supplementary material**

Hou, et,al. DRG Payment Does Not Predispose to Negative Clinical Outcomes in General Surgery Cases: Evidence from a Tertiary Hospital in China

Supplementary Table S1. The number and ratio of patients with nodal metastasis and lateral neck dissection in KD19

|  | Before  2019.3-2022.2 | After  2022.3-2025.2 | p |
| --- | --- | --- | --- |
| Number of patients | 9343 | 8600 |  |
| Number of neck nodal metastasis, n (%) | 2772 (29.6%) | 3612 (42.0%) | < 0.001 |
| Number of lateral neck dissection, n (%) | 1863 (19.9%) | 2750 (31.9%) | < 0.001 |
